# Transcriptomic and Histological Analysis of the Response of Susceptible and Resistant Cucumber to *Meloidogyne incognita* Infection Revealing Complex Resistance *via* Multiple Signaling Pathways

**DOI:** 10.3389/fpls.2021.675429

**Published:** 2021-06-14

**Authors:** Xvzhen Li, Yinhui Sun, Yuting Yang, Xiaopei Yang, Wanyu Xue, Meiqian Wu, Panpan Chen, Yiqun Weng, Shuxia Chen

**Affiliations:** ^1^College of Horticulture, Northwest A&F University/Shaanxi Engineering Research Center for Vegetables, Yangling, China; ^2^United States Department of Agriculture, Agriculture Research Service, Vegetable Crops Research Unit, Horticulture Department, University of Wisconsin, Madison, WI, United States

**Keywords:** cucumber, transcriptome analysis, defense responses, *Cucumis sativus*, *Cucumis metuliferus*, *Meloidogyne incognita*

## Abstract

The root-knot nematode (RKN), *Meloidogyne incognita*, is a devastating pathogen for cucumber (*Cucumis sativus* L.) specially in production under protected environments or continuous cropping. High level RKN resistance has been identified in African horned melon *Cucumis metuliferus* (CM). However, the resistance mechanism remains unclear. In this study, the comparative analysis on phenotypic and transcriptomic responses in the susceptible cucumber inbred line Q24 and the resistant CM, after *M. incognita* infection, was performed. The results showed that, in comparison with Q24, the CM was able to significantly reduce penetration numbers of second stage juveniles (J2), slow its development in the roots resulting in fewer galls and smaller giant cells suggesting the presence of host resistance in CM. Comparative transcriptomes analysis of Q24 and CM before and after *M. incognita* infection was conducted and differentially expressed genes (DEGs) associated with host resistance were identified in CM. Enrichment analyses revealed most enriched DEGs in Ca^2+^ signaling, salicylic acid (SA)/jamonate signaling (JA), as well as auxin (IAA) signaling pathways. In particular, in CM, DEGs in the Ca^2+^ signaling pathway such as those for the calmodulin and calcium-binding proteins were upregulated at the early stage of *M. incognita* infection; genes for SA/JA synthesis/signal transduction were markedly activated, whereas the IAA signaling pathway genes were inhibited upon infection suggesting the importance of SA/JA signaling pathways in mediating *M. incognita* resistance in CM. A model was established to explain the different molecular mechanisms on *M. incognita* susceptibility in cucumber and resistance to *M. incognita* infection in CM.

## Introduction

The root-knot nematode (RKN), *Meloidogyne incognita*, is among the most destructive pathogens of many crop plants. Nematodes use a hollow protrusible stylet to break into the cells of host roots to induce highly specialized nematode feeding sites (NFS), withdraw nutrients, and inhibit the plant immune system (Abad and Williamson, 2010; Siddique and Grundler, 2018). A large number of galls are formed on the host roots, the nutrition balance in the host plant is disrupted resulting in significant yield reduction (Kayani et al., 2017). Globally, annual losses caused by the RKNs are estimated to be over $118 billion (Atkinson et al., 2012).

As a warm-season vegetable, cucumber (*Cucumis sativus* L.) is mainly cultivated in greenhouses or plastic tunnels in China. In recent years, RKN and other soil-borne diseases have become a serious constraint in cucumber production (Gine et al., 2014; Mao et al., 2016). Host resistance against *M. incognita* has been identified in some wild relatives in *Cucumis* like *C. metuliferus* Naud, *C. hystrix* Chakr, and *C. melo* var. *texanus* (Faske, 2013; Expósito et al., 2018; Cheng et al., 2019). Among them, upon infection of *M. incognita* on the resistant *C. metuliferus*, fewer J2 (second-stage juveniles) nematodes were able to penetrate in the roots, form smaller giant cells (GCs); the nematodes grow slower and produce fewer eggs than on susceptible plants (Faske, 2013; Ye et al., 2017).

The defense responses induced by plant-parasitic nematodes are associated with both pattern-triggered immunity (PTI) and effector-triggered immunity (ETI) (Teixeira et al., 2016). The tomato resistance gene *Mi-1.2* (*R* gene) is involved in PTI, which is associated with the nematode recognition and signal transduction pathway, and ETI is also related to defense responses and mitogen-activated protein kinase (*MAPK*) signaling cascades (Liu et al., 2016); the defense responses induced by nematodes are associated with Ca^2+^ signaling and *WRKY*, which both consists of positive regulators of plant defense transcriptional networks (Davies et al., 2015; Manosalva et al., 2015).

Researchers also believe that phytohormones might play an important role in the plant defense against nematode infection, such as salicylic acid (SA), jasmonic acid (JA), auxin (IAA) and the responsive signaling pathway (Gheysen and Mitchum, 2019). SA and JA pathway are activated when plants are infected by RKNs, leading to a hypersensitive response (HR) and systemic acquired resistance (SAR) (Nahar et al., 2011; Molinari et al., 2014). During the parasitic stage of RKN, the initiation and maturation of NFS are associated with the local accumulation of IAA (Siddique and Grundler, 2018). The expression network of defense-related genes functioning in nematode resistance currently includes IAA and cell cycle-related genes in cucumber (Wang et al., 2018), phenylpropanoid biosynthesis in cucumber (Ye et al., 2017), metabolite defense signaling pathways and hormones in tomato (Shukla et al., 2018), transcription factors and hormones in sweet potato (Lee et al., 2019), and defense-related genes in tobacco (Li et al., 2018). Therefore, the resistance mechanism of host to RKN involves in complex and multiple metabolic pathways.

While no cucumber cultivars with high resistance to root knot nematodes in China, the identification of host resistance in *C. metuliferus* presents us an opportunity to understand the phenotypic and molecular mechanisms of host resistance against this pest. Thus, in this study, we conducted microscopic and histological investigation in the susceptible cucumber inbred line Q24 and a resistant CM upon *M. incognita* infection. We also conducted transcriptome profiling in the two lines before and after infection, and identified differentially expressed genes (DEGs) and defense responsive pathways associated with host resistance.

## Materials and Methods

### Plant Materials and *M. incognita* Inoculum Preparation

The cultivated cucumber inbred line Q24 (Chinese Long, north China fresh market type) and an inbred of cucumber wild relative, *Cucumis metuliferus* (CM) were used in this study. CM was a derivative of PI 482443 through self-pollination for at least three generations. The germinated seeds were sown in 8 × 8 cm plastic pots with autoclaved sands. All pots were arranged in an illumination incubator (RXZ-5COB-LED, Ningbo, Zhejiang, China) under conditions of 26°C, 14 h-light/18°C, 10 h-dark, and RH 85–90%. The seedlings were watered with 1/2 Hoagland’s nutrient solution twice weekly.

*M. incognita* were maintained on the susceptible tomato cultivar “Dongfen No. 3” in a glasshouse at 22–26°C. The roots of plants were cut and sterilized with 0.5% NaOCl, and then rinsed with distilled water (dH_2_O). The eggs were collected using a 25-μm sieve and kept for hatching in double layered paper tissues in a Petri dish with dH_2_O at 28°C for 24 h (Fudali et al., 2013). J2s were re-suspended in dH_2_O, and the concentration was adjusted to 1,000 J2’s per mL for inoculation.

### Comparative Analysis of *M. incognita* Development in Resistant and Susceptible Lines

Q24 and CM seedlings with two fully expanded true leaves were inoculated by dropping 1 mL J2s suspension into a 2 cm deep hole that was 1 cm away from the seedling. The control plants were watered with 1 mL dH_2_O.

The roots at 3 days post inoculation (dpi) were isolated from Q24, CM and stained with acid fuchsin according to Zhang et al. (2017) to count the number of J2s under a stereoscopic fluorescence microscope (MZ10F-Leica, Germany). For the developmental stages of the *M. incognita* within the galls were observed at 25 dpi, the numbers of nematodes at J3, J4, female and male were recorded in the roots of CM Q24 and CM. For each sample, there were four biological replicates (3 seedlings per replicates) and three technical replicates.

The number of galls on roots was counted at 25 dpi. The galls were also photographed using a stereoscopic fluorescence microscope (MZ10F-Leica, Germany) to measure the gall size under the microscope using the SPOT software (SPOT Imaging, United States). Twenty-four seedlings were used for every biological replicate, and the experiment was repeated in three times.

To observe the cellular changes induced by *M. incognita* infection, paraffin sections of Q24 and CM roots were prepared according to Wang et al. (2018). Root samples at both 3 and 15 dpi were fixed in FAA, transferred into a graded ethanol series (10–100%) and chloroform series (10–100%), and followed by embedding in paraffin. The samples were cut into 8-μm-thick sections, stained with hematoxylin eosin, sealed with gum, and observed under a fully automatic upright fluorescence microscope (OLYMPUS BX63).

### Expression Analysis by qRT-PCR

The expression patterns of 4 genes related to hormone metabolism were analysis with quantitative real-time PCR (qPCR). The qPCR primers of the four genes are provided in Supplementary Table 1. Total RNAs were extracted from the roots of Q24 and CM at 2, 3, 4, 5 dpi using the RNA plus kit (TaKaRa Biotechnology, Dalian, China). cDNA synthesis was performed with the RevertAid First Strand cDNA Synthesis Kit (Roche Diagnostics, Indianapolis, United States). qPCR was performed with SYBR green (TaKaRa Biotechnology, Dalian, China) on a QuantStudio5 real time-PCR machine (Life Technologies, United States). Expression values were normalized to the *CsUBQ5* (Csa2G301530) gene. Reactions were carried out using two biological, and three technical replicates for each sample.

### Transcriptome Profiling and Enrichment Analysis

For RNA-Seq, roots of *M. incognita*-inoculated (T, 3 dpi) and control (CK) plants of Q24 and CM were selected and collected, gently washed, flash frozen in liquid nitrogen and stored at −80°C. The roots of three plants were used as one replicate and three replicates are included (total 12 samples). Total RNA isolation, cDNA synthesis/library construction, and sequencing were all conducted through commercial service at the Novogene Inc. (Beijing, China). The cDNA samples were further enriched by PCR to construct the final cDNA libraries that were sequenced using the Hiseq 2500 (150 bp paired-ends) sequencing platform.

High quality, clean reads were aligned to the 9930 cucumber reference genome (v2.0)^1^ using HISAT2 (v2.0.5). Differentially expressed genes (DEGs) in CM-T, CM-CK, Q24-T, and Q24-CK pairs were identified using the DESeq2 R package (1.16.1). A DEG in a particular comparison was defined as the adjusted *P* < 0.05 and—log2FoldChange—>0.0. All DEGs were subjected to enrichment analysis including Gene Ontology (GO) and Kyoto Encyclopedia of Genes and Genomes (KEGG), which were conducted in the ClusterProfiler R package (V.3.4.4). Additional analysis was performed for DEGs involving in plant-pathogen interactions (PPI), hormone signaling, and signal transduction in host defense responses. RNA-seq raw data are available under the website of NCBI^2^ with the Bioproject ID PRJNA707668.

### Statistical Analysis of Data

All numerical data collected from this study were subjected to statistical analysis and significance tests. Specifically, physiological data measured upon nematode infection were analyzed by one-way analysis of variance (ANOVA), and statistical significance was determined by pairwise comparisons (*P* < 0.05), which was implemented in SPSS 22.0 (Statistical Package for the Social Sciences, Chicago, IL, United States). The expression patterns of DEGs were showed the value of the log2FoldChange and presented as a heat map by using MeV software (MultiExperiment Viewer 4.7.4).

## Results

### Comparison of Symptom and Gall Development in the Roots of Q24 and CM in Response to *M. incognita* Inoculation

**FIGURE 1:**
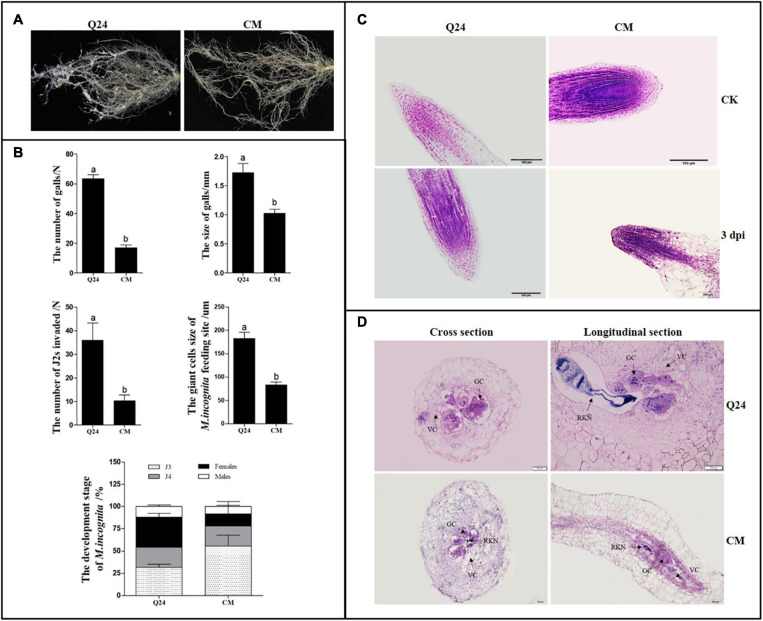
Symptom development and growth on susceptible Q24 and resistant CM plants. **(A)** The roots of Q24 and CM at 25 dpi; **(B)** comparison of disease indexes of Q24 and CM at 25 dpi. Error bars indicate the standard error of the means. Different letters on the bars “a, b, c” indicate statistically significant means between Q24 and CM at 25 dpi at *P* < 0.05 based on Duncan’s *t*-tests; **(C)** root tips of Q24 and CM at 3 dpi and the corresponding controls; **(D)** cross and longitudinal sections of nematode feeding sites (NFS) inside the galls at 15 dpi. GC, giant cell; VC, vacant cell; V, vacuole.

The galls formed on the roots of the susceptible Q24 and resistant CM were counted and measured. In the susceptible Q24, there were significantly more and larger galls that were observed throughout the root system than in CM, where the small galls were clustered mainly on some lateral roots (Figure 1A). At 25 dpi, on average, 63 and 17 galls on roots of Q24 and CM, which were 1.73 and 1.03 mm in size, respectively (Figure 1B).

The microscopic root structures in the two lines were comparing by histological examination before and after *M. incognita* infection. In roots of control, there were no visible differences in root tip cell structure between Q24 and CM; the root crown was intact with regular arrangement of root tip cells. At 3 dpi, loose cells and void tissue in the apical meristem zone of Q24 were observed, whereas those in CM remained compact (Figure 1C). At 15 dpi, much larger GCs and more nuclei in GCs were observed in Q24 roots than that in CM, and GCs connected with parenchyma cells were observed in Q24, which may facilitate supply of nutrients to GCs from the surrounding parenchyma cells. In contrast, fewer GCs were observed in CM; there were also few nuclei in the GCs. Hollow cells around the GCs in the roots of CM were observed, which separated GCs from parenchymal cells. This may result in difficulty for GCs to obtain nutrients from the surrounding parenchyma cells (Figure 1D). The GC size in CM was significantly smaller than that in Q24 (Figure 1B). Taken together, these results suggested host resistance in CM plant plays an important role in reducing the severity of symptom development although the CM plant is not immune to *M. incognita infection.*

### Number Invasion and Development Difference of *M. incognita* in Roots of Resistant and Susceptible Plants

The number and development of nematodes were compared in the roots of CM and Q24. The significant difference in nematode invasion was observed between Q24 and CM at 3 dpi, on average, 36 and 10 J2s, respectively. The J2s invaded into the roots of CM was hindered to some extent. In Q24 at 25 dpi, after J2s invasion, 33.6% of J2s developed to adult females and 11.9% developed to adult males. The J2s development in CM roots was obviously different from J2s in roots of Q24, which were only 13.7% females and 8.4% males at 25 dpi. The development of most J2s in CM was blocked to J3 and J4 stages. These observations indicated that, few J2s were able to penetrate into roots of CM at the stage of infection and J2s were developing poorly in roots of CM at the parasitic stage (Figure 1B).

### Relative Expression of Selected Genes Related to Defense Response and Hormone Metabolism

In order to determine the expression time of genes induced by *M. incognita* infection, the expression patterns of several genes were validated in the roots of Q24 and CM at 2, 3, 4, 5 dpi. Among them, *MAPK9* (Csa2G361890) was known to be involved in plant-pathogen interaction, phenylalanine ammonia-lyase genes (*PAL*, Csa6G445760), *bZIP* transcription factor *TGA7* (Csa2G403160) were related to SA and indole-3-acetic acid-amido synthetase GH3 (*GH3.6*, Csa6G125240) were related to IAA. It showed that *MAPK9*, *TGA7* was significantly downregulated after *M. incognita* infection and *GH3.6* and *PAL* were upregulated significantly at 3 dpi in Q24. In CM after infection, the *TGA7*, *PAL* were significantly upregulated at 3 dpi and then *TGA7* downregulated at 4, 5 dpi. The *MAPK9* was significantly downregulated at 3 dpi and gradually return to expression at 5 dpi. The expression of *GH3.6* in CM was low and changed little after *M. incognita* infection (Supplementary Figure 1).

### Transcriptional Sequencing of Cucumber Plants in Response to *M. incognita*

Based on the expression pattern of genes selected in qPCR in Q24 and CM after infection, 3 dpi and the CK were selected for transcriptome analysis. Approximately 47.47–67.57 million 150 bp paired-end clean reads per sample were obtained after cleaning and checking. Approximately 97% of clean reads were aligned uniquely to the cucumber genome using the software HISAT2 v2.0.5 (Supplementary Table 2). The correlation clustering among the three biological replicates of each sample was conducted based on the expression level of all genes. All biological replicates showed correlation coefficients above 0.9 indicating good reproducibility between biological replicates (Supplementary Figure 2).

### Differential Gene Expression Analysis and Functional Categorization

**FIGURE 2:**
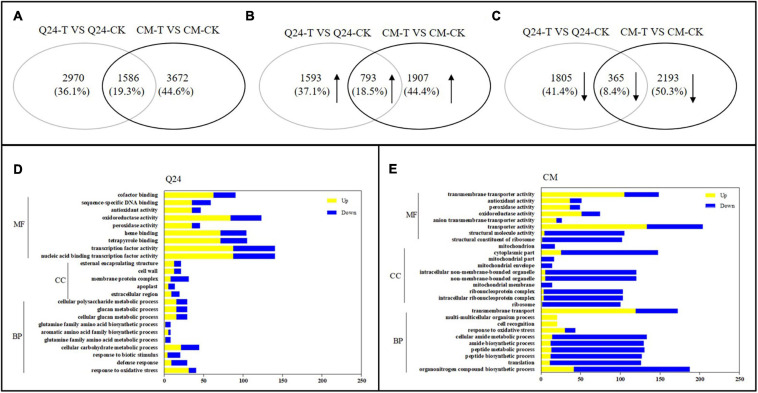
The number of differentially expressed genes (DEGs) and Gene Ontology (GO) enrichment analysis in responding to the infection of *M. incognita* in Q24, CM. **(A)** All DEGs mapped in Q24 and CM (adjusted *P* < 0.05); **(B)** the upregulated DEGs mapped in Q24 and CM (adjusted *P* < 0.05); **(C)** the downregulated DEGs mapped in Q24 and CM (adjusted *P* < 0.05). The percentage in **(A–C)** was the number of DEGs related to the total of identified DEGs in all DEGs, in all upregulated DEGs or in all downregulated DEGs in Q24, CM; **(D)** GO terms of DEGs in Q24; **(E)** GO terms of DEGs in CM. Yellow indicated upregulation and blue indicated downregulation. DEGs were classified into specific biological process categories with high classification stringency (adjusted *P* < 0.05). The horizontal ordinate represents the number of genes in the category. MF, molecular function; CC, cellular component; BP, biological process.

A total of 17,612 and 17,966 genes were identified by the DESeq2 R package among inoculated samples compared with respective non-inoculated controls of Q24 and CM, respectively (false discovery rate < 0.03, DESeq2 padj < 0.05 and | log2FoldChange| > 0.0). There were 4,556 significantly differentially expressed genes (DEGs) between Q24 and the control, and 5,258 DEGs were identified between CM and the control. From these, there were 1,586 DEGs identified in both genotypes (Figure 2A). Among the identified DEGs, 2,386 DEGs and 2,700 DEGs were significantly upregulated in Q24 and CM, respectively, and 793 were upregulated in both genotypes (Figure 2B). There were 2,170 DEGs significantly downregulated in Q24 and 2,558 DEGs significantly downregulated in CM. 365 DEGs were downregulated in both Q24 and CM after *M. incognita* infection (Figure 2C). Among these DEGs, *MAPK9* (Csa2G361890), *PAL* (Csa6G445760), *TGA7* (Csa2G403160), and *GH3.6* (Csa6G125240) were all identified among inoculated samples compared with respective controls of Q24 and CM. The expression of these four genes in transcriptomic were in accordance with the results of qRT-PCR (Supplementary Figure 3).

Based on GO enrichment analysis, the categories of total DEGs were enriched significantly in biological terms in both phenotypes in response to *M. incognita* (adjusted *P* < 0.05). In comparison of 3 dpi with the control of Q24, GO biological enriched significantly in the categories of response to stimulus and signaling, secondary metabolites metabolic processes and biosynthetic processes, protein related with cell wall and membrane synthesis process, transcription factor activity, reduction and scavenging of reactive oxygen species (ROS) products, and more genes showing upregulation than downregulation in these terms (Figure 2D). In resistant plants of CM, more GO biological terms were enriched than that in Q24, including translation process, organonitrogen compound metabolic process, peptide biosynthetic process and metabolic process, transmembrane transport, transporter activity and so on. Comparing the DEGs in each biological term, more DEGs were identified in CM than that in Q24, and most of them were downregulated in each term, all of them indicating that a large number of genes played a vital role in CM responding to *M. incognita* (Figure 2E).

**FIGURE 3:**
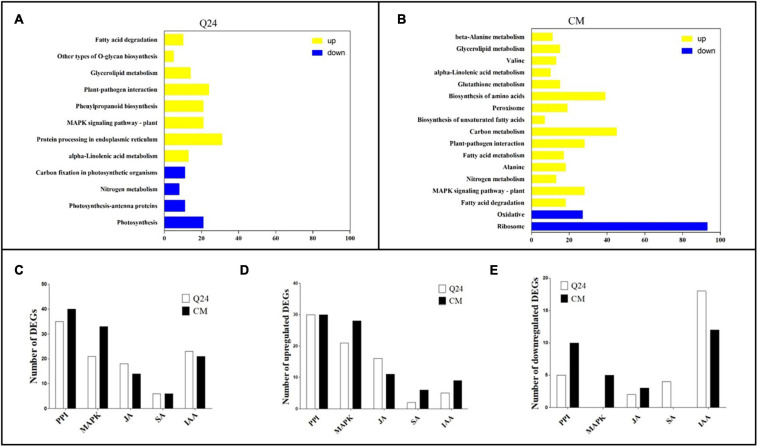
Kyoto Encyclopedia of Genes and Genomes (KEGG) enrichment analysis in responding to the *M. incognita* infection of Q24, CM. DEGs were classified into pathway categories with high classification stringency (adjusted *P* < 0.05). The horizontal ordinate represents the number of genes in the category. **(A)** KEGG terms of DEGs in Q24; **(B)** KEGG terms of DEGs in CM. Yellow indicated upregulation and blue indicated downregulation; **(C)** the DEGs mapped to hormone metabolism and plant pathogen interactions of Q24 and CM responding to infection (adjusted *P* < 0.05); **(D)** the number of upregulated DEGs and downregulated DEGs mapped to hormone metabolism and plant pathogen interactions of Q24 (adjusted *P* < 0.05); **(E)** the upregulated DEGs and downregulated DEGs mapped to hormone metabolism and plant pathogen interactions of CM (adjusted *P* < 0.05). PPI, plant-pathogen interactions; MAPK, mitogen-activated protein kinase signaling cascades.

On the basis of the KEGG Pathway database, the DEGs enrichment were analyzed using clusterProfiler R package to identify the metabolic pathways in which they function (adjusted *P* < 0.05). In comparison of infection with the control of Q24, the enrichment of upregulated biological process was involving PPI, phenylpropanoid biosynthesis, MAPK signaling pathway and protein processing in endoplasmic reticulum. Furthermore, a small part of genes was enriched in downregulated process including photosynthesis, nitrogen metabolism and carbon fixation in photosynthetic organisms (Figure 3A). Comparing with the biological process of Q24 enrichment, more biological process and more DEGs were involved in the response of resistant plant CM to *M. incognita*, such as upregulated biological process MAPK signaling pathway, PPI, Carbon metabolism, Biosynthesis of amino acids and downregulated process Ribosome (Figure 3B). The difference in metabolic pathways between Q24 and CM indicated that the mechanisms were different in two genotypes facing to *M. incognita* infection.

Most researchers believed that genes related to nematode recognition and phytohormones metabolism might play an important role in the plant defense against RKN infection. So multiple DEGs were related to PPI, MAPK signaling cascades, hormone biosynthesis and signal transduction were analysis in this study. The number of DEGs related to PPI was greatest between the treatment and its control; 35 DEGs were identified in Q24, and 40 were identified in CM. The second largest number of DEGs were related to MAPK signaling cascades, which was consisting of 21 DEGs in Q24 and 33 in CM (Figure 3C). Between the two genotypes and their controls, more than half of the DEGs related to PPI, MAPK signaling cascades, JA pathway were upregulated, while most of the DEGs related to IAA were downregulated. DEGs related to MAPK signaling cascades and SA showed a significant difference between the two genotypes and their controls. Most DEGs related to SA were downregulated in Q24 and upregulated in CM (Figures 3D,E).

### Differentially Expressed Genes Related to Ca^2+^ and MAPK Signaling

**FIGURE 4:**
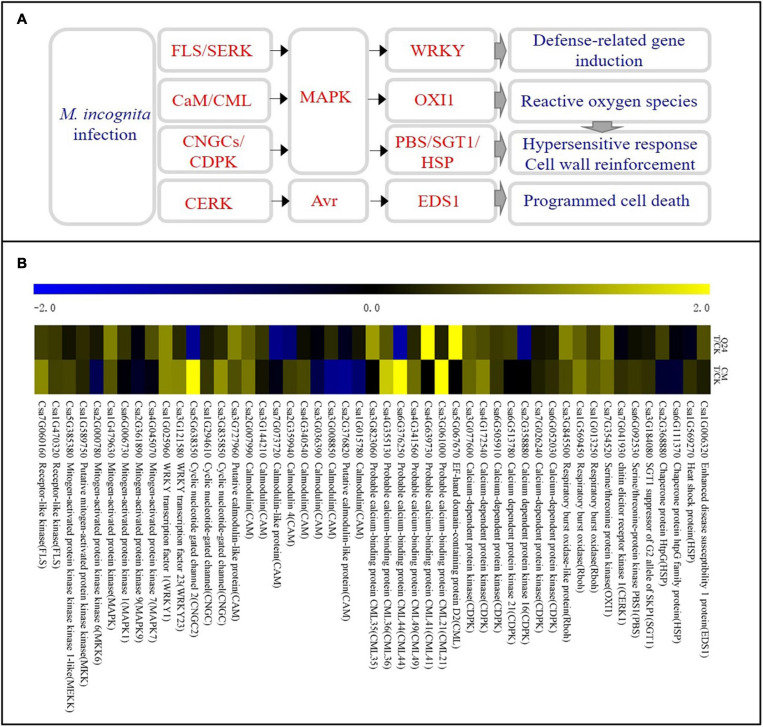
Ca^2+^ signaling pathway and heat map of differentially expressed genes in Q24 and CM responding to infection. **(A)** All differentially expressed genes mapped to Ca^2+^ signaling of Q24 and CM responding to infection; **(B)** the heat map showed the value of the log2FoldChange of genes compared with that of the control in plant defense in Q24 and CM. Yellow indicated up expression and blue indicated down expression.

Some DEGs were involved in the plant defense were identified in the cucumber plant after *M. incognita* infection (Figure 4A). Most of them were involved in the Ca^2+^ and MAPK signaling pathways and were significantly upregulated at the early stage of *M. incognita* infection, and there was a notable difference between Q24 and CM. The calcium-binding protein genes (*CML*, Csa5G067670, Csa4G639730, Csa3G823060), calmodulin gene (*CaM*, Csa3G727960), *MAPK* (Csa1G479630), serine/threonine protein kinase (*OXI1*), respiratory burst oxidase (*Rboh*) were identified clearly upregulated in Q24, which indicating that the defense response of Q24 to *M. incognita* mainly referred to signal transduction upon CaM/CML, later the activation of defense response genes and the outbreak of ROS. While the defense response of CM to *M. incognita* referred to signal transduction upon pathogen perception through not only CaM/CML, but also cyclic nucleotide gated channel (CNGCs)/calcium-dependent protein kinase (CDPK), such as *CNGC* (Csa3G835850, Csa5G638350) and *CDPK* (Csa6g052030) were upregulated in CM. Additionally, more genes involved in the defense response genes, HR and cell wall reinforcement were also induced in CM than in Q24, such as WRKY transcription factor 23 (*WRKY23*, Csa3G121580), chitin elicitor receptor kinase 1(*CERK1*, Csa7G041930), Serine/threonine-protein kinase (*PBS1*, Csa6G092530), SGT1 suppressor of G2 allele of SKP1(*SGT1*, Csa3G184080). All of them indicated that the activation of defense response genes, HR, cell wall reinforcement, ROS and programmed cell death in CM were induced after *M. incognita* infection (Figure 4B).

### Differentially Expressed Genes Related to the SA Biosynthesis and Signaling Pathway

**FIGURE 5:**
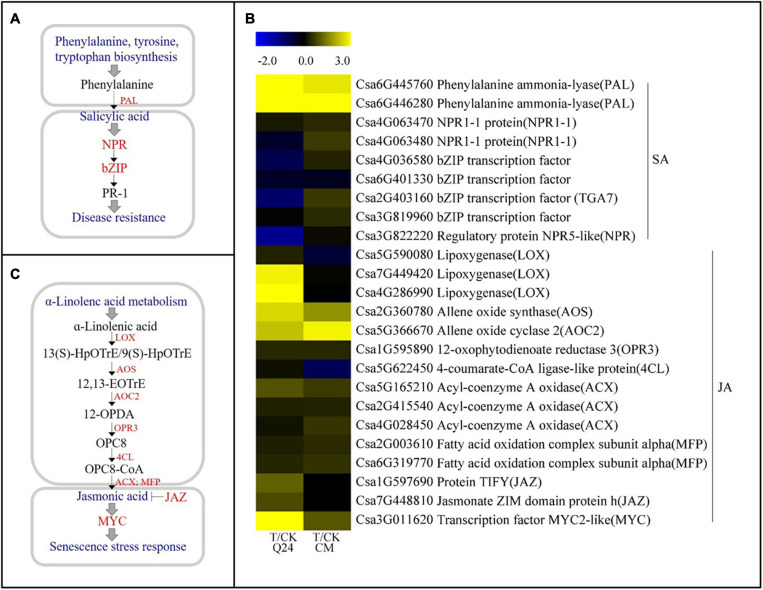
SA and JA biosynthesis and signal transduction pathways and heat map of differentially expressed genes in Q24 and CM responding to infection. **(A)** SA biosynthesis and signal transduction; **(B)** JA biosynthesis and signal transduction; **(C)** the heat map showed the value of the log2FoldChange of genes compared with that of the control in SA and JA pathway in Q24 and CM. Yellow indicated up expression and blue indicated down expression.

Three gene families associated with the SA pathway were identified both in Q24 and CM in response to *M. incognita* infection (Figure 5A), including *PAL*, regulatory protein gene NPR (*NPR1-1*), and bZIP transcription factor (*bZIP/TGA*). The *PAL* genes (Csa6G445760, Csa6G446280), which encode a critical enzyme in the SA biosynthesis pathway, were significantly upregulated both in Q24 and CM. On the other hand, the *NPR1-1* (Csa4G063470), which is a key regulator of the SA signaling pathway, and the bZIP transcription factors (Csa4G036580, Csa2G403160, Csa3G819960) were not induced or significantly downregulated in Q24 and they were highly induced in CM after infection (Figure 5B). The SA biosynthesis and signaling pathways were markedly activated in CM.

### Differentially Expressed Genes Related to the JA Biosynthesis and Signaling Pathway

Some molecules associated with the JA biosynthesis and signaling pathway were identified after *M. incognita* infection (Figure 5C). Most genes upstream of JA biosynthesis were highly induced after infection, particularly in Q24, such as the lipoxygenase gene (*LOX*, Csa7G449420, Csa4G286990), allene oxide synthase (*AOS*, Csa2G360780), and allene oxide cyclase 2 (*AOC2*, Csa5G366670). While some key genes participated in both JA biosynthesis and JA-dependent SAR, maintaining a low level of expression in Q24 and induction in CM during *M. incognita* infection, such as the acyl-coenzyme a oxidase *(ACX*, Csa4G028450) encoding key enzymes for acyl-CoA oxidase, and the fatty acid oxidation complex subunit alpha genes (*MFP*, Csa2G003610, Csa6G319770) encoding a multifunctional protein in JA biosynthesis. Additionally, the negative regulatory factors in the JA signaling pathway, *JAZs* (Csa1G597690, Csa7G448810), were significantly induced in Q24 and not changed in CM at 3 dpi (Figure 5B).

### Differentially Expressed Genes Related to the IAA Biosynthesis and Signaling Pathway

**FIGURE 6:**
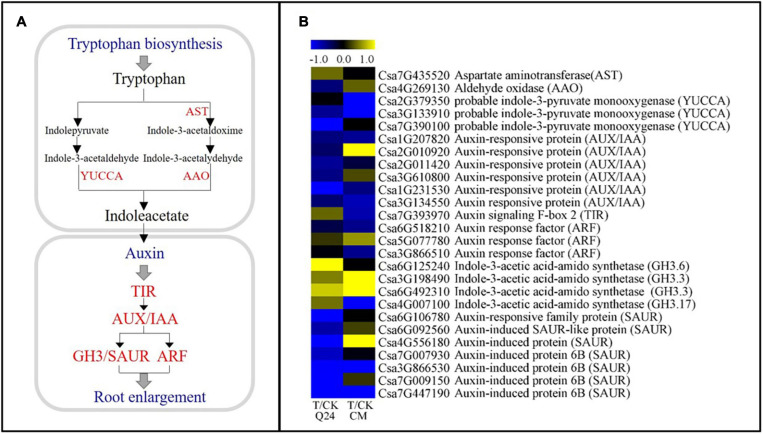
IAA biosynthesis and signal transduction pathways and heat map of differentially expressed genes in Q24 and CM. **(A)** IAA biosynthesis and signal transduction pathway; **(B)** the heat map showed the value of the log2FoldChange of genes compared with that of the control in IAA pathway. Yellow indicated up expression and blue indicated down expression.

IAA has been reported to be involved in the formation of GCs. In this study, IAA-related DEGs showed significant differences between Q24 and CM (Figure 6A). Many major auxin-related genes were significantly downregulated in CM at 3 dpi, including the indole-3-pyruvate monooxygenase (*YUCCA*, Csa2G379350, Csa3G133910), auxin signaling F-box 2 (*TIR*, Csa7G393970), auxin response factor (*ARF*, Csa6G518210, Csa3G866510), *GH3* (*GH.3.17*, Csa4G007100), which were the key gene in the TAA-YUC pathway of auxin biosynthesis and auxin signaling pathways. While the genes of *TIR* (Csa7G393970) and *GH3* (Csa6G125240, Csa3G198490, Csa6G492310, Csa4G007100) were upregulation in Q24 after nematode infection, indicating that the IAA signal was transduced and affected the GC formation in Q24. Several genes related to IAA signal transduction were also found upregulated in CM, such as auxin-responsive protein genes *AUX/IAA* (Csa2G0010920) and *GH3* (Csa3G198490, Csa6G492310), auxin-induced protein gene (*SAUR*, Csa2G010920), which indicated that the invasive J2s interfered with signal transmission in the NFS of CM (Figure 6B). These results indicated that auxin biosynthesis and signaling pathways were inhibited more strongly in CM than in Q24.

## Discussion

### The Plant Defense Response Was Activated by *M. incognita* Infection Upon Ca^2+^ Signaling

Ca^2+^/calmodulin has long been considered a crucial component in the mediating plant defense against various biotic attackers, and cellular Ca^2+^ fluxes are among the earliest detectable biochemical features upon pathogen recognition (Reddy et al., 2011). It was proved that Ca^2+^ signaling is then triggered and translated by CaM, CMLs, calcineurin-like proteins (CBLs), and CDPKs in order to interact with effector proteins of plant-parasitic nematodes (Perochon et al., 2011; Haegeman et al., 2012). Ca^2+^ signaling mediators that also induced PTI, ETI and participated in SA- or JA-mediated long-term resistance to pathogens. In addition, the sequential activation of the MAPK cascade eventually leads to activation of the expression of specific genes and, subsequently, the induction of HR, ROS, cell wall reinforcement and defense-related gene expression (Bigeard et al., 2015). Other studies have shown that the resistance of gene *R_*Mc*__1(__*blb)*_* in potato to *Meloidogyne chitwoodi* were dependent on a hypersensitive response and involves calcium, indicating that Ca^2+^ plays a role in the *R_*Mc*__1_*_(__*blb)*_-mediated resistance against *M. chitwoodi* in potato (Davies et al., 2015). Calcium/calmodulin-mediated signaling might coordinate various regulatory pathways in response to *Heterodera glycine* infection in soybeans (Zhang et al., 2017). We speculate that Ca^2+^ mediated regulation might function as a dispatcher between MAPK cascade, PTI, ETI, SA-, and JA-mediated resistance to response to *M. incognita* infection. Therefore, the differences in DEGs of Ca^2+^ signals between Q24 and CM may mediate different regulatory pathways to respond to *M. incognita* infestation, which requires further research.

### SA and JA Signal Pathway Activation Was Involved in the Basal Defense of CM Against *M. incognita* Infestation

At the early stage of J2 infection, chorismate mutase was secreted and suppressed plant immunity by regulating the SA pathway (Wang et al., 2018). Studies have shown that increasing SA levels enhance the resistance of host plant against RKN infection (Priya et al., 2011). The bZIP transcription factor is a transcriptional co-activator of NPR1 in the transduction of SA, and loss-of-function studies have shown that class II TGA factors (bZIP transcription factor) in particular play important roles in SA-mediated gene expression. The tga2/tga5/tga6 triple mutant shows compromised PR-1 expression (Zhang et al., 2003), indicating that the SA transduction signal was activated in CM during the early infection stage. Therefore, we assumed that the basic defense mechanisms of CM were activated by the upregulated expression of *bZIP* and *NPR* related to SA signal transduction during *M. incognita* infection.

The functions of the genes in the JA metabolic pathway were more complex during the process of nematode infection. Many studies have shown that JA acts as a defense factor against nematode in plants (Nahar et al., 2011; Zhou et al., 2015), and overexpression of *OsAOS* in rice enhances resistance to *M. graminicola* (Mei et al., 2006). The *Arabidopsis* mutant *lox3-1*, *lox4-1*, and *aoc* are all more susceptible to RKN infection (Ozalvo et al., 2014; Naor et al., 2018). It has also been reported that JA-related genes in GCs are suppressed, as verified by transcriptome analyses (Ji et al., 2013). Our results showed that the expression of most genes related to JA biosynthesis were not significantly induced in the susceptible Q24 compared with the resistant CM. In addition, two alleles of *JAZ*, a negative regulator of the JA signaling pathway, were significantly induced in Q24 but suppressed in CM after *M. incognita* infection, indicating that JA played a positive role in the defense of the resistant germplasm against *M. incognita*.

### Suppression of the Expression of Genes Related to IAA Limited the Development of GCs in CM

GCs formation is a key factor for a successful plant-nematode interaction after the nematode arrive into the cortical cylinder. If GCs appear as degenerated, the nematode development or reproduction will be suppressed (Expósito et al., 2018). Studies have shown that the GCs in feeding site of *C. metuliferus* were smaller and less voluminous, with fewer nuclei than susceptible plants and some of them were empty of cytoplasm along with a slow nematode development (Ye et al., 2017; Expósito et al., 2018, 2020). In our study, smaller galls and GCs and fewer nuclei in the GCs of CM than Q24 were observed, and the J2s development in CM roots was significantly slower in comparison to that in Q24, which was consistent with previous reports (Wang et al., 2018).

IAA is the key factor for NFS formation (Perrot-Rechenmann, 2010). The cell wall growth and cell cycle activation of GCs was promoted by IAA biosynthesis and response genes in the host cell after nematode infection (Hwang and Williamson, 2003). GH3, an auxin-responsive promoter, was rapidly and transiently activated during root gall initiation by *Meloidogyne* (Hutangura et al., 1999). Other studies showed that the nematode development was impaired in auxin insensitive mutants because of the arrest in early feeding cell formation (Goverse et al., 2000; Gheysen and Mitchum, 2009). Moreover, the expression of most homologous genes of auxin-responsive promoter *GH3* were inhibited in the resistance material cucumber introgression line “IL10–1” (Wang et al., 2018). In the present study, fewer members of the *GH3* gene family were found upregulated in resistant CM than in susceptible Q24, which was consistent with previously published reports. In addition, the upregulation of *AUX/IAA*, the auxin/indole-3-acetic acid transcriptional repressor gene, and the downregulation of *TIR* in resistant CM after *M. incognita* infection, indicating that the IAA concentration was significantly lower in resistant CM than that in the control. Thus, we conjectured that the lack of auxin contributed greatly to the abnormal development of GCs in CM, which was consistent with the in resistant line “IL10–1” (Wang et al., 2018).

## Conclusion

**FIGURE 7:**
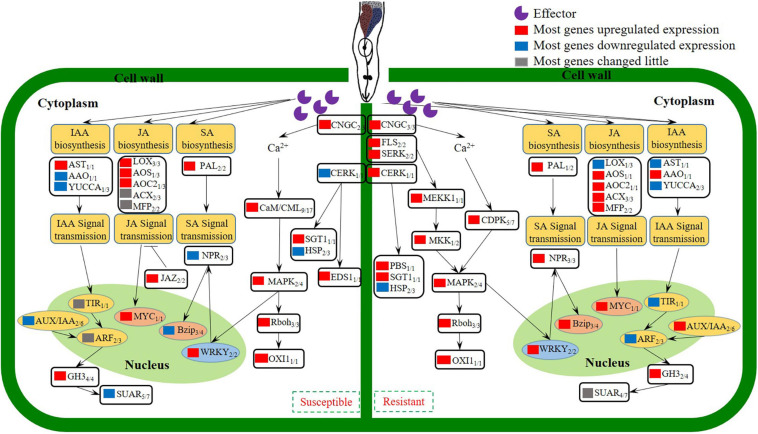
Model depicting how the coordinated signaling network in cucumber leads to the induction of local and systemic defense against *M. incognita* invasion.

In conclusion, studies on an inbred of cucumber wild relative CM identified with *M. incognita* resistance, indicated that reducing J2s invasion, suppressing the development of J2s and the GCs of *M. incognita* were the resistance characteristics. Comparison of transcriptomes revealed that Ca^2+^ signaling, SA/JA genes were activated, which triggered an active defense response that leading to the resistance of CM against *M. incognita*. Transcriptomes also revealed that IAA genes were inhibited in CM, which caused the abnormal development of GCs, and finally resulted in the blocking of *M. incognita* development. According to our data, a model was established to probe the changes of gene expression involved in the biological processes of *M. incognita* recognition, signal transduction, hormone biosynthesis and signal transmission in different resistant species (Figure 7). A number of genes involved in the recognition and signaling of nematode infestation in CM were identified, thus providing a basis for research examining the interaction between cucumber plants and *M. incognita*.

## Data Availability Statement

The original contributions presented in the study are publicly available. This data can be found here: NCBI repository, accession: PRJNA707668.

## Author Contributions

SC designed the experiment and revised the manuscript. YW participated in the data analysis and preparation of the manuscript. XL performed the experiments and wrote the manuscript. YS conducted the RNA-Seq. YY performed the sampling for transcriptome analyze. XY carried out the qRT-PCR. WX, MW, and PC contributed to the reagents and materials. All authors have read and approved the final manuscript.

## Conflict of Interest

The authors declare that the research was conducted in the absence of any commercial or financial relationships that could be construed as a potential conflict of interest.

## Footnotes

1 http://cucurbitgenomics.org/organism/2/

2 https://www.ncbi.nlm.nih.gov/

## Supplementary Material

Supplementary Figure 1 Relative expression level of selected hormone metabolism related genes in roots of Q24 and CM at 2, 3, 4, 5 dpi by qPCR. *MAPK9*, mitogen-activated protein kinase 9; *PAL*, phenylalanine ammonia-lyase genes; *TGA7*, bZIP transcription factor; *GH3.6*, indole-3-acetic acid-amido synthetase GH3.6.

Supplementary Figure 2 The heat map showing the correlation between all root samples of CM and Q24 at 3 dpi and the control. The color represents the correlation coefficient between every two samples. The deeper the color, the greater the correlation coefficient.

Supplementary Figure 3 The FPKM of 4 genes related hormone metabolism in transcriptomic data. *MAPK9*, mitogen-activated protein kinase 9; *PAL*, phenylalanine ammonia-lyase genes; *TGA7*, bZIP transcription factor; *GH3.6*, indole-3-acetic acid-amido synthetase GH3.6.

Supplementary Table 1 Primers used in this study.

Supplementary Table 2 The statistics of RNA-seq sequencing data in this study. CM-ck, Q24-ck and CM-T, Q24-T represent the control and the treatment of CM and Q24. 1, 2, 3 represent three biological replicates.
