# Supplementary figures and images for: Transcriptomic and Histological Analysis of the Response of Susceptible and Resistant Cucumber to Meloidogyne incognita Infection Revealing Complex Resistance via Multiple Signaling Pathways
Source: Front Plant Sci. 2021 Jun 14;12:675429. doi: 10.3389/fpls.2021.675429 (PMC8236822; doi:10.3389/fpls.2021.675429)

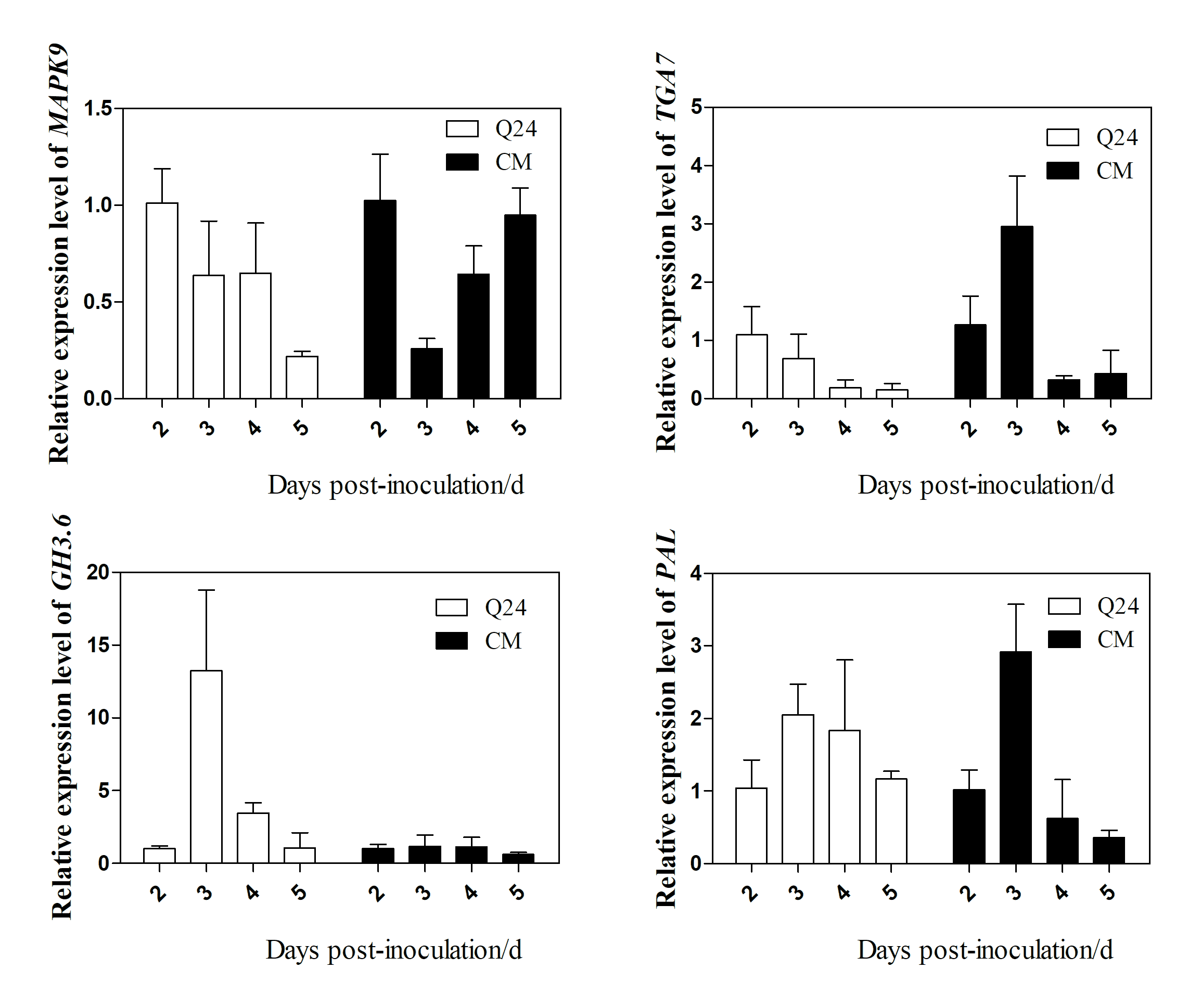

Supplement: Supplementary Figure 1 — Relative expression level of selected hormone metabolism related genes in roots of Q24 and CM at 2, 3, 4, 5 dpi by qPCR. MAPK9, mitogen-activated protein kinase 9; PAL, phenylalanine ammonia-lyase genes; TGA7, bZIP transcription factor; GH3.6, indole-3-acetic acid-amido synthetase GH3.6. [file Image_1.TIF]

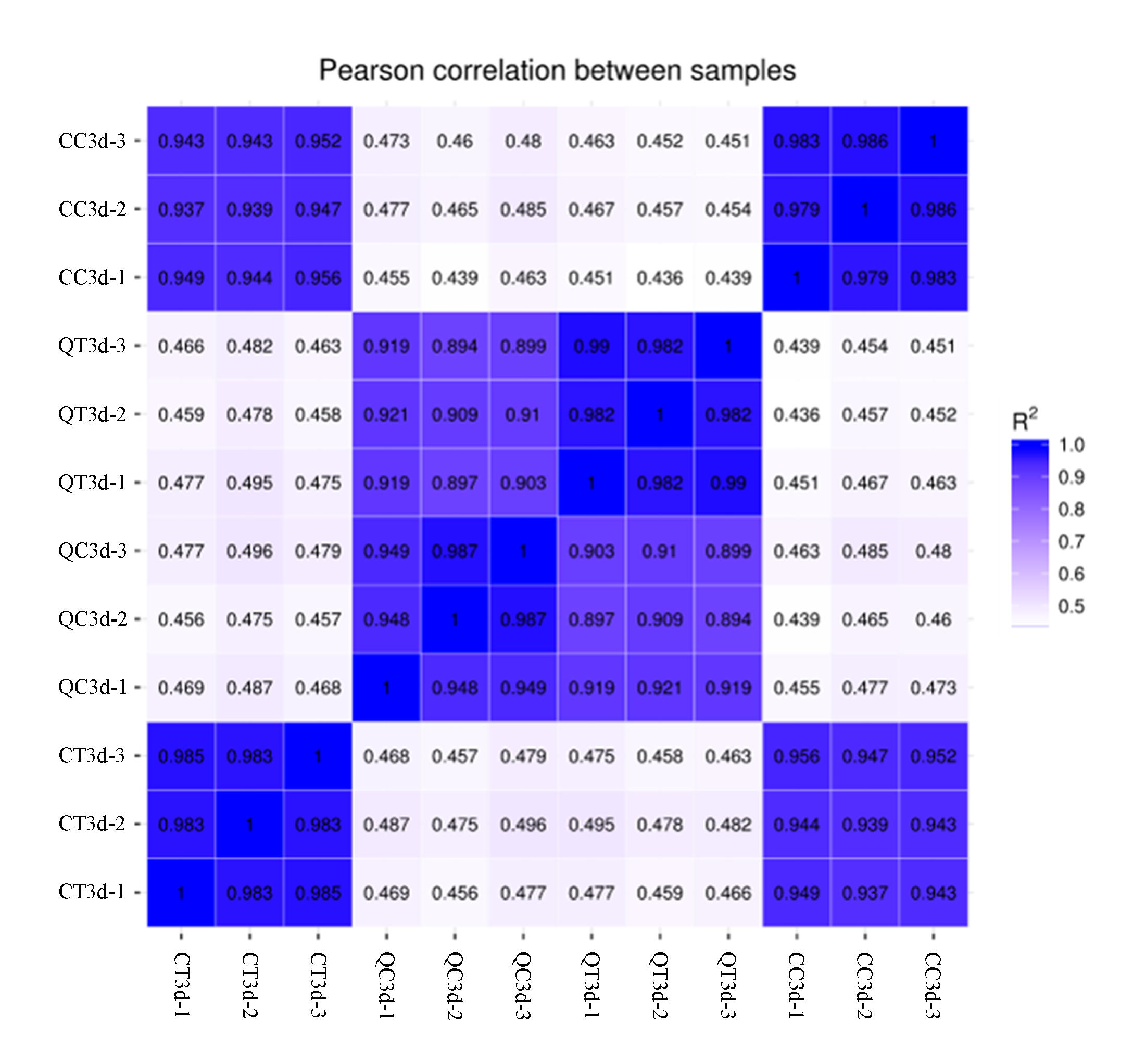

Supplement: Supplementary Figure 2 — The heat map showing the correlation between all root samples of CM and Q24 at 3 dpi and the control. The color represents the correlation coefficient between every two samples. The deeper the color, the greater the correlation coefficient. [file Image_2.TIF]

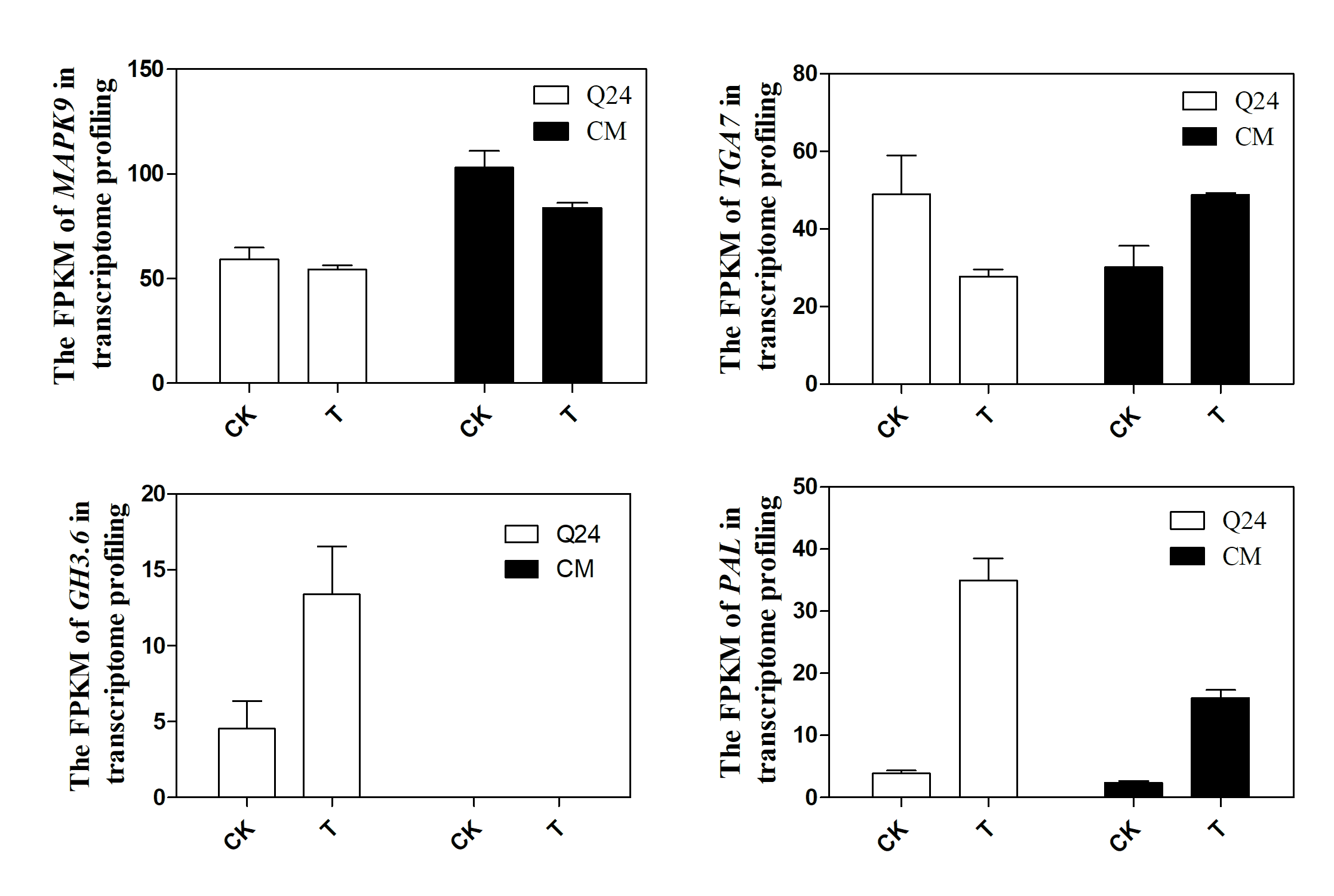

Supplement: Supplementary Figure 3 — The FPKM of 4 genes related hormone metabolism in transcriptomic data. MAPK9, mitogen-activated protein kinase 9; PAL, phenylalanine ammonia-lyase genes; TGA7, bZIP transcription factor; GH3.6, indole-3-acetic acid-amido synthetase GH3.6. [file Image_3.TIF]
